# Postoperative behavior of thoracolumbar/lumbar curve and coronal balance after posterior thoracic fusion for Lenke 1C and 2C adolescent idiopathic scoliosis

**DOI:** 10.1186/1748-7161-10-S1-O67

**Published:** 2015-01-19

**Authors:** Masayuki Ishikawa, Kai Cao, Long Pang, Kota Watanabe, Mitsuru Yagi, Masafumi Machida, Yasuyuki Fukui, Morio Matsumoto

**Affiliations:** 1 Spine and Spinal Cord Center, Mita Hospital, International University of Health and Welfare, Japan; 2 Department of Orthopedic Surgery, School of Medicine, Keio University, Japan; 3 Department of Orthopedic Surgery, Murayama Medical Center, Japan

## Objectives

Controversy still exists in surgical strategy for primary thoracic and compensatory lumbar curves in adolescent idiopathic scoliosis (AIS). Benefit of selective thoracic fusion for this curve type is spontaneous lumbar curve correction with saving of more lumbar mobile segments. However, a risk of postoperative coronal decompensation after selective thoracic fusion has also been reported. This multicenter retrospective study was conducted to evaluate postoperative behavior of thoracolumbar/lumbar (TLL) curve and coronal balance after posterior thoracic fusion for Lenke 1C and 2C AIS.

## Material and methods

Twenty-four Lenke 1C and 2C AIS patients who underwent posterior thoracic fusion were included in this study. Mean age and Risser grade of patients at time of surgery were 15.7 years old and 3.5, respectively. Mean follow-up period was 29.6 months. Surgical procedures were PS construct with the lowest instrumented vertebra (LIV) ending at L3 or above.

Investigated clinical data were number of fused vertebrae, and level of LIV. Preoperative and postoperative radiographic measurements were performed on Cobb angles of MT and TLL curves, LIV tilt and coronal balance. The gap differences between LIV and reference vertebrae (EV, NV, SV, Apex of TLL curve) were counted (LIV-EV, LIV-NV, LIV-SV, ApexTLL-LIV, respectively). Factors related to final Cobb angle of TLL curve and postoperative change of coronal balance were detected.

## Results

Mean number of fused vertebrae was 9.4, and LIV were T11 in seven, T12 in eight, L1 in five, L2 in three, and L3 in one. Mean Cobb angles for MT and TLL curves were 59.0 degrees and 43.7 degrees preoperatively, and were corrected to 21.5 degrees (64.2% correction) and 22.0 degrees (49.4% correction) at final follow-up, respectively. Mean LIV tilt was 20.5 degrees preoperatively, and was corrected to 9.4 degrees at final follow-up. Mean coronal balance was -5.6mm preoperatively and was corrected to -14.6mm at final follow-up. The gap differences of LIV-EV, LIV-NV, LIV-SV, and ApexTLL-LIV averaged 0.9, 0.9, 0.5, and 2.0, respectively.

Final Cobb angle of TLL curve was positively correlated to immediate postoperative Cobb angle of MT curve and LIV tilt. Postoperative change of coronal balance was positively correlated to LIV-SV and preoperative coronal balance.

## Conclusion

Spontaneous correction of TLL curve occurred consistently by correcting MT curve and making LIV more horizontal after posterior thoracic fusion for Lenke 1C and 2C AIS. The more distal fixation to SV resulted in coronal balance shifting to the more left postoperatively.

